# Gut metagenomic features of frailty

**DOI:** 10.3389/fcimb.2024.1486579

**Published:** 2024-11-25

**Authors:** Zharkyn Jarmukhanov, Nurislam Mukhanbetzhanov, Elizaveta Vinogradova, Samat Kozhakhmetov, Almagul Kushugulova

**Affiliations:** Center for Life Sciences, National Laboratory Astana, Nazarbayev University, Astana, Kazakhstan

**Keywords:** frailty, gut microbiome, metagenomics, metabolic pathways, microbial diversity

## Abstract

This study investigates the relationship between frailty severity and gut microbiome characteristics in adults in Kazakhstan. We analyzed 158 participants across four frailty severity (mild to very severe) using metagenomic sequencing of stool samples. Frailty was significantly correlated with age, weight, and functional measures like walking speed and grip strength. Microbial diversity decreased significantly with increasing frailty. Beta diversity analysis revealed distinct clustering patterns based at phylum level. Taxonomically, we observed a significant inverse correlation between Firmicutes abundance and frailty. Classes like Clostridia and Erysipelotrichia decreased with frailty, while Bacteroidia and Actinobacteria increased. At the family level, Oscillospiraceae showed a positive correlation with frailty. Functionally, we identified significant correlations between frailty measures and specific metabolic pathways. The frailty index negatively correlated with pathways involved in cobalamin, arginine and molybdenum cofactor biosynthesis and positively correlated with folate biosynthesis. Physical performance measures strongly correlated with pathways related to nucleotide biosynthesis, and one-carbon metabolism. We propose these identified features may constitute a “frailty-associated metabolic signature” in the gut microbiome. This signature suggests multiple interconnected mechanisms through which the microbiome may influence frailty development, including modulation of inflammation, alterations in energy metabolism, and potential impacts on muscle function through microbial metabolites.

## 1 Introduction

Frailty is a condition characterized by reduced physiological resilience and increased risk of adverse health outcomes. Recently, frailty has been shown to be a better predictor of health outcomes in older adults than chronological age. As the global population ages, understanding the underlying mechanisms of frailty becomes increasingly crucial for developing effective interventions and improving the quality of life for older adults. Central to the pathophysiology of frailty is altered metabolic activity. Frailty is associated with dysregulation of multiple metabolic pathways, including energy metabolism, protein synthesis, and oxidative stress responses (Picca et al., 2018). These metabolic alterations contribute to the hallmarks of frailty, such as sarcopenia, inflammation, and reduced physiological reserve (Grosicki et al., 2018).

Recent studies have started to shed light on the potential role of the gut microbiome in frailty (Strasser and Ticinesi, 2023). The human gut microbiome, which consists of various microorganisms, has been implicated in influencing the risk of age-related chronic diseases and syndromes, though its specific role in frailty is not well-understood (Morales et al., 2022; Zhu et al., 2023; He et al., 2024; Yang et al., 2024). This knowledge gap is concerning because frailty is closely tied to the body’s overall metabolic activity, which is linked to the health of the intestinal microbiome.

While some studies have explored aspects of the frailty-microbiome relationship, such as the connection between frailty and reduced fecal microbiota biodiversity, as well as the decreased abundance of certain types of bacteria, these studies only scratch the surface of this complex relationship (Lim et al., 2021; Xu et al., 2021). Similarly, while some studies have identified specific bacterial differences between frail and healthy individuals, the functional implications of these differences remain largely unknown (Xu et al., 2021; Chen et al., 2022).

Preliminary research has delved into potential mechanisms connecting the gut and frailty, such as changes in intestinal barrier integrity, systemic inflammation, and metabolic dysregulation. For example, a study found that a gut microbiota metabolite could induce frailty-like symptoms in mice (Chen et al., 2022). However, these findings, while promising, highlight how much more there is to learn about the intricate relationship between the microbiome and frailty.

This study aims to analyze the relationship between frailty severity and microbiome characteristics. The goal is to identify specific frailty signatures in the microbiome, which could be a crucial step in understanding the complex interplay between frailty, metabolism, and the gut microbiome, potentially leading to new avenues for frailty prevention and treatment.

## 2 Materials and methods

### 2.1 Study population

The Cohort Study examines health and frailty across Kazakhstan’s urban and rural populations. The National Laboratory Astana’s Local Ethics Committee (Protocol 05-2023, dated 21.11.2023, IORG0006963) and University Medical Center Local Ethics Committee (Protocol 3/2023/ПЭ, dated 14.07.2023) approved the research. All 158 participants provided informed consent. Participants ranged in age from 39 to 87. Physical examinations assessed vital signs, anthropometric measurements, and mobility. Stool samples for microbiome analysis were collected using DNA/RNA Shield Fecal Collection Tubes from Zymo Research (R1101).

### 2.2 Frailty index score

Frailty Index Score (FIS) – consists of several physical stress tests. We used a battery of tests consisting of standing up from a chair for a while, walking a certain area for a while and measuring the grip strength of both hands. Rising from a chair was measured in seconds, and the respondents were asked to stand up from a seated position and sit back down five times as quickly as possible. In the next test, participants were instructed to walk a distance of 2 meters twice at their usual pace. The time to complete each 2-meter walk was measured in seconds, and the average of the two trials was recorded. The maximum grip strength of both hands was measured using a handheld dynamometer. The test was performed twice for each hand, and the highest value for each hand was recorded in kilograms. All tests were conducted by trained interviewers who used standardized protocols and calibrated equipment. Times for the Chair Stand and Walking Speed tests were measured using a digital stopwatch. Results from all three tests were recorded on standardized checklists for each participant. The FIS was then calculated using a validated algorithm that incorporates the results from these three tests, with weightings assigned to each component based on their relative importance in predicting adverse health outcomes. This composite score provides a comprehensive assessment of physical frailty, encompassing lower body strength, mobility, and upper body strength.

### 2.3 Metagenomic research

DNA was extracted using the ZymoBiomics DNA Microprep kit (Cat. No: D4301), and DNA concentrations were measured using the Nanodrop 2000/2000c (ThermoFisher). Sterile water was used as a negative control. Following the standard Illumina protocols, the samples were sequenced at Novogene (Beijing, China) on the Illumina NovaSeq6000 platform. Each metagenomic sample yielded an average of 6 Gb of raw sequencing data. Data processing and analysis were conducted on a Dell Precision 7920 workstation equipped with dual Intel Xeon Gold 6230 processors (80 cores each at 2.10 GHz), 252 GB of DDR4 RAM, a 10 TB SSD for fast primary storage, and a 4 TB HDD for additional backup. The HUMAnN 3.0 pipeline was employed for data processing. Knead data v0.12.0 was used to clean metagenomic data, remove contaminating sequences such as ribosomal RNAs and host contaminants, and improve data quality before analysis. UniRef90 was used for protein sequence annotation and retrieval. Default parameters were chosen to ensure standard analysis and optimal compatibility across the various stages of data processing. Taxonomic profiling was conducted using the MetaPhlAn 4 database, specifically version mpa_vJan21_CHOCOPhlAnSGB_202103, incorporating updated markers and enhanced taxonomic coverage. The mean sequencing depth was determined to be 28.347 million reads per sample.

### 2.4 Bioinformatic and statistical analysis

The data analysis was conducted using Python version 3.9.16 and R version 4.2.2. We excluded taxonomic and functional features that appeared in less than 70% of the samples to power the underlying association analyses. For the differential analysis, we used MaAsLin2 version 1.12.0 and included relevant covariates (age, body mass index). To account for multiple tests, we applied the Benjamini-Hochberg false discovery rate (FDR-BH) correction method. For FDR-corrected values in models adjusting for covariates, the significance level was set at 0.25. Between-class diversity was assessed using Bray-Curtis and Jaccard indices at the species and phylum level using scikit-bio 0.6.2. Baseline characteristics were compared using ANOVA, Kruskal-Wallis H test, or Chi2 test as appropriate, and correlations were assessed using Spearman’s r coefficient using SciPy v1.13.1. The correlation between the Frailty Index (FI) and Age and FI and Body Mass Index (BMI) was calculated using Spearman’s rank correlation coefficient. Adonis2 function from the vegan 2.6-4 package was used to assess the significance of clustering in decomposition, while accounting for covariates. Within-sample diversity was assessed using the Observed, Shannon, and Simpson indices. LEfSe utils were used for cladogram construction.

### 2.5 Bioinformatic and statistical analysis

The data analysis was conducted using Python version 3.9.16 and R version 4.2.2. We excluded taxonomic and functional features that appeared in less than 70% of the samples to power the underlying association analyses. For the differential analysis, we used MaAsLin2 version 1.12.0 and included relevant covariates (age, body mass index). To account for multiple tests, we applied the Benjamini-Hochberg false discovery rate (FDR-BH) correction method. For FDR-corrected values in models adjusting for covariates, the significance level was set at 0.25. Between-class diversity was assessed using Bray-Curtis and Jaccard indices at the species and phylum level using scikit-bio 0.6.2. Adonis2 function from the vegan 2.6-4 package was used to assess the significance of clustering in decomposition, while accounting for covariates. Within-sample diversity was assessed using the Observed, Shannon, and Simpson indices. LEfSe utils were used for cladogram construction.

## 3 Results

### 3.1 Participant characteristics and frailty distribution

**Table 1** presents the characteristics of the research participants in the frailty groups. Among the 158 participants, 6 (3.8%) were classified as mildly frail (Frailty Index Score 0.20-0.49), 14 (8.9%) as moderately frail (0.50-0.74), 94 (59.5%) as severely frail (0.75-1.29), and 44 (27.8%) as very severely frail (≥1.30). The distribution showed that the majority of participants had severe (n = 94, 59.5%) or very severe frailty (n = 44, 27.8%), with a smaller number in the mild (n = 6, 3.8%) and moderate (n = 14, 8.9%) frailty groups. Frailty was significantly correlated with age (r=0.34, p<0.000013), weight (r=0.32, p<0.000043), Speed (r=0.39, p<0.000009), and Strength Tests (r=-0.33, p<0.000462). At the same time, Frailty was significantly associated with Speed Test (r=0.3295, p=0.012) and less significantly with Strength Test (r=-0.2001, p=0.092) while adjusting for age and weight. Gender and habitat were not significantly associated with Frailty.

**Table 1:** Baseline characteristics.

Parameters	Mild (n=3)	Moderate (n=21)	Severe (n=87)	Very severe (n=47)	Method, p=value
**Age, M ± Sd, years**	49.5 ± 6.5	52.9 ± 10.5	55.3 ± 8.1	62.6 ± 11.7	one-way ANOVA, p = 3.74e-05
**Sex, f/m (n=f/m)**	3/3 (n=1/3)	12/2 (n=10/11)	68/26 (n=68/19)	38/6 (n=42/5)	Chi2, p = 0.0976
**BMI, M ± Sd**	23.4 ± 3.1	26.8 ± 3.9	28.8 ± 6.6	32.6 ± 4.8	Kruskal-Wallis H-test, p = 1.94e-05
**Urban, n (%)**	1 (16,7)	5 (35,7)	27 (28,7)	5 (11,4)	Chi2, p = 0.1012
**Rural, n (%)**	5 (83,3)	9 (64,3)	67 (71,3)	39 (88,6)	
**Speed, M ± Sd**	11.8 ± 4.4	15.9 ± 3.6	15.2 ± 5.1	20.8 ± 8.4	Kruskal-Wallis H-test, p = 0.00021
**Strength, M ± Sd**	135.6 ± 42.6	118.4 ± 31.3	132.9 ± 44.8	105.5 ± 29.5	Kruskal-Wallis H-test, p = 0.051

f/m, female/male; M±Sd - M, mean; SD, standard deviation; n, number.

This age-frailty correlation supports the established idea that frailty risk increases with age. Notably, our results closely match those of Kojima et al. (2018), who reported a correlation between frailty and age in their meta-analysis (Kojima et al., 2018).

The positive correlation with weight contributes to the ongoing discussion on body composition and frailty. While frailty is often linked to weight loss, our results are consistent with research by Hubbard et al. (2010) suggesting that obesity can also contribute to frailty through increased inflammation and reduced physical function (Hubbard et al., 2010).

Additionally, we found significant associations between frailty and functional assessments: a strong positive correlation with the Speed Test and a moderate negative correlation with the Strength Test.

### 3.2 Taxonomic analysis of the gut microbiome

**Figure 1:**
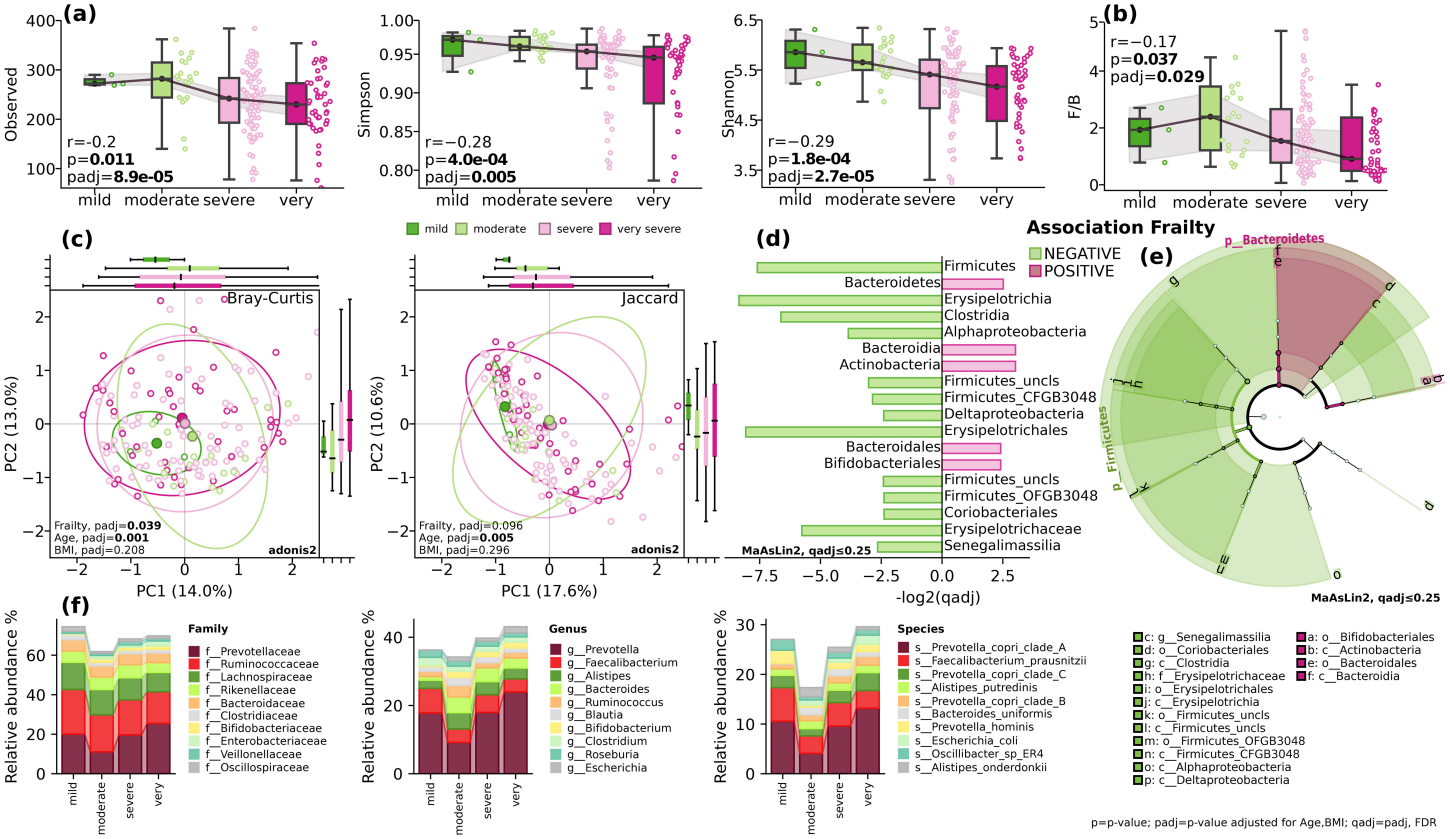
Gut microbial diversity and composition. **(A)** alpha-diversity. Observed, Simpson and Shannon indices; **(B)** The Firmicutes/Bacteroidetes ratio (F/B). Spearman’s r (p) and GLM (padj); **(C)** beta diversity. Bray-Curtis and Jaccard indices. Adonis2, 999 permutations; **(D)** Barplot of qadj values for differentially distributed taxa, MaAsLin2, FDR, q ≤ 0.25; **(E)** cladogram depicting the phylogenetic distribution of differentially abundant microbiota. The central point marks the root of the tree and extends to lower taxonomic levels from phylum to species. Built using LEfSe utils for MaAsLin2, FDR, q ≤ 0.25; **(F)** stack plots showing the mean relative abundance at taxonomic levels of family, genus and species. BMI=body mass index; p=p-value; padj=p-value adjusted for age and BMI; qadj= padj, FDR.

According to Fried et al., frailty is defined as a clinical syndrome characterized by the presence of three or more of the following criteria: unintentional weight loss, self-reported exhaustion, weakness, reduced gait speed, and low physical activity (Fried et al., 2001). We investigated association between alpha diversity indices Observed (p=0.55, padj=0.24), Simpson (p=0,004, padj=0.06), Shannon (p=0,011, padj=0.06), and Pielou (p=0,002, padj=0.06) and frailty while adjusting for covariates (**Figure 1A**). All taxonomic evenness indices showed a statistically significant decrease with increasing frailty, while absolute richness remained comparable. Interestingly, we observed an even more pronounced decrease in taxonomic evenness at the strain level (Simpson padj=0.1, Shannon padj=0.035, Pielou, padj=0.032).

The analysis of beta diversity using Bray-Curtis and Jaccard indices showed modest differences in clustering patterns at phylum level (PERMANOVA, Bray-Curtis, F=2.2515, padj=0.019), but not at species level (PERMANOVA, Bray-Curtis, F=1.0275, padj=0.34) while adjusting for covariates (**Figure 1C**).

When looking at the taxonomic composition, while controlling for the effect of age and body mass index, at the phylum level, there was a significant inverse correlation between the relative abundance of Firmicutes and the frailty index (p<0.018, padj=0.06) and positive correlation with Bacteroidetes (padj=0.07, qadj=0.14) and Actinobacteria (padj=0.13, qadj=0.22) (**Figures 1D–F**). Analysis at the class level revealed a decrease in the relative abundance of Clostridia (p=0.02, qadj=0.14) and increase of Bacteroidia (padj=0.055, qadj=0.22). The decrease in mobility assessed in Speed Test was linked to a decrease in the relative abundance of Erysipelotrichia (p=8.4e-04, padj=0.035), Alphaproteobacteria (p=0.054, padj=0.16), and Deltaproteobacteria (p=0.053, padj=0.16) classes (**Figures 1D–F**). At the order level, a significant decrease was found with Erysipelotrichales (p=8.4e-04, padj=0.021). While at the family level, Oscillospiraceae showed a positive correlation with decrease in mobility (padj=0.013, qadj=0.23). On the other hand, the Strength-test assessment demonstrated negative associations at the order level with Bacteroidales (p=0.07, qadj=0.23), Eggerthellales (padj=0.051, qadj=0.23), and Erysipelotrichales (padj=2.0e-04, qadj=0.005) (**Figures 1D–F**). Changes in the relative abundance of these bacteria and striking decline in diversity, as illustrated in **Figure 1**, suggest shifts in energy metabolism, nutrient absorption, gut barrier function, and systemic inflammation, all of which may contribute to frailty.

### 3.3 Functional analysis of the gut microbiome

Our study found significant connections between the frailty index and specific metabolic pathways, shedding new light on the relationship between frailty and microbial metabolism. We observed significant correlations between the frailty index and several key metabolic pathways, as well as we identified significant correlations between specific metabolic pathways and individual components of the frailty index, such as speed and strength tests (**Supplementary Table 1**). The decrease in mobility assessed in Speed Test demonstrated negative correlation with energy metabolism (padj=8.6e-04, qadj=0.11), Superpathway of Polyamine Biosynthesis II (padj=0.002, qadj=0.15) and molybdenum cofactor biosynthesis (padj=5.1e-04, qadj=0.11) and positive correlation with Deoxyribonucleotides de Novo Biosynthesis (padj=0.009, qadj=0.21).

Particularly noteworthy are the strong correlations between physical performance measures (speed and strength tests) and pathways related to nucleotide biosynthesis, one-carbon metabolism. These associations indicate that better physical function is linked to a more robust and metabolically active gut microbiome, aligning with recent studies on the gut-muscle axis in aging (Ticinesi et al., 2019).

These findings are consistent with recent research on the gut-muscle axis in aging, which suggests a bidirectional relationship between gut microbiota and muscle health (Grosicki et al., 2018; Picca et al., 2018). The dysbiosis observed in frail individuals may contribute to sarcopenia and decreased physical function, further exacerbating the frailty syndrome (Thevaranjan et al., 2017).

## 4 Discussion

We believe that the imbalance of microorganisms observed in frail individuals leads to changes in metabolic activities within the gut microbiome. These changes ultimately results in the frailty phenotype. They manifest in declines in physical performance, such as reduced speed and overall physical function (Ticinesi et al., 2019). Our study has identified connections between frailty and specific patterns in the gut microbiome in adults. As frailty becomes more severe, there is a noticeable decrease in microbial diversity. This observation aligns with recent studies suggesting that a loss in microbial diversity is a characteristic of frailty, potentially leading to systemic inflammation and metabolic dysregulation (Ghosh et al., 2020; Larson et al., 2022; Lim and Nam, 2023).

The analysis provides resolution of metabolic capabilities across multiple taxonomic levels, particularly within Firmicutes subgroups, with detailed quantitative correlations (**Supplementary Table 2**, **Supplementary Figure S1**). Most notably, energy-related pathways (pyruvate fermentation, methanogenesis, and respiration) show well-documented strong negative correlations with Bacteroidetes and positive correlations with Firmicutes. Beyond these established patterns, our comprehensive analysis uniquely revealed coordinated relationships across 23 pathways, including novel associations in molybdenum cofactor biosynthesis and polyamine metabolism.

We observed a notable decrease in Firmicutes and Erysipelotrichia an increase in Bacteroidetes. These shifts in microorganisms and overall diversity can be linked to impaired gut barrier integrity, increased oxidative stress, and heightened inflammatory responses, all contributing to frailty (Oh et al., 2022).

Our findings also suggest that the increased production of pyrimidine deoxyribonucleotides by the microbiome could contribute to frailty due to the essential role of these nucleotides in DNA synthesis and repair. As DNA repair mechanisms become less efficient with age, the increased demand for these nucleotides might reflect compensatory mechanisms that, when overwhelmed, contribute to cellular senescence and frailty (Lim and Nam, 2023).

A reduced production of polyamines by the microbiome also appears to play a role in frailty. Polyamines are critical for cellular resilience, regenerative capacity, and autophagy—a process that becomes increasingly impaired with age. Lower levels of polyamines may contribute to the inflammatory and degenerative processes observed in frailty (Larson et al., 2022).

Our observation of decreased activity in the Superpathway of Polyamine Biosynthesis II (padj=0.002, qadj=0.15) alongside reduced mobility highlights the critical biological roles of polyamines, like spermidine and spermine, in maintaining health and influencing frailty development. Polyamines support gut barrier integrity and immune regulation; hence, reduced production might compromise gut health, increasing permeability and systemic inflammation, key frailty characteristics (Hirano et al., 2021; Soda, 2022). This aligns with our finding of decreased *Firmicutes*, which aid in polyamine production.

Additionally, polyamines contribute to cellular stress resistance and longevity by inducing autophagy and mitigating oxidative stress (Hirano et al., 2021; Soda, 2022). Their reduction could hasten cellular aging. Polyamines also have anti-inflammatory effects, suppressing pro-inflammatory cytokines and modulating immune functions. Thus, decreased polyamine biosynthesis may exacerbate chronic low-grade inflammation associated with frailty, linking gut microbiome changes to physical decline.

We also noted increased deoxyribonucleotide biosynthesis (padj=0.009, qadj=0.21) with decreased mobility, suggesting a compensatory response to DNA damage or stress. This shift, alongside reduced polyamine production, reflects broader metabolic dysfunction in frailty affecting repair mechanisms and inflammation.

Our findings highlight the importance of considering microbial metabolic functions, rather than focusing solely on taxonomic composition, in studying the role of the gut microbiome in frailty (Kim and Jazwinski, 2018). This functional perspective is supported by recent literature advocating for a shift towards understanding the metabolic capabilities of the microbiome as a more accurate predictor of health outcomes in ageing populations (O’Toole and Jeffery, 2018; Larson et al., 2022; Lim and Nam, 2023). Such an approach could lead to the development of microbiome-based diagnostics and therapeutics aimed at preventing or mitigating frailty.

While our study focused on the relationship between frailty, age, weight, and functional measures, it’s important to acknowledge that frailty is a complex, multifactorial syndrome. Other factors known to influence frailty include chronic diseases, nutritional status, physical activity levels, socioeconomic factors, and environmental exposures. These factors may also impact gut microbiome composition and function, potentially mediating or moderating the microbiome-frailty relationship we observed. Future studies incorporating these additional factors alongside microbiome data could provide more comprehensive insights into the complex interplay between host factors, the gut microbiome, and frailty development. This multifaceted approach could lead to more targeted interventions for frailty prevention and management.

The study has certain limitations and include its cross-sectional design, precluding causal inferences, and the lack of functional validation for identified metabolic pathways. We did not collect data on participants’ medical history, medication use, nutrition, physical activity, or socioeconomic status—factors that could influence both frailty and gut microbiome composition. The sample size and gender distribution may limit statistical power for certain associations. Future longitudinal studies with larger, diverse cohorts should incorporate these factors and employ functional approaches like metabolomics to elucidate causal relationships between gut microbial changes and frailty progression. Such comprehensive investigations could inform targeted interventions for frailty prevention and management.

## 5 Conclusions

In conclusion, our study provides a characterization of gut microbiome signatures associated with frailty, offering new insights into the complex interplay between gut microbiota, metabolism, and frailty. These findings provide foundation for innovative strategies in the prevention, diagnosis, and management of frailty in aging populations.

## Data Availability

The datasets presented in this study can be found in online repositories. The names of the repository/repositories and accession number(s) can be found in the article/**Supplementary Material**.
