# Supplementary figures and images for: Gut metagenomic features of frailty
Source: Front Cell Infect Microbiol. 2024 Nov 25;14:1486579. doi: 10.3389/fcimb.2024.1486579 (PMC11625779; doi:10.3389/fcimb.2024.1486579)

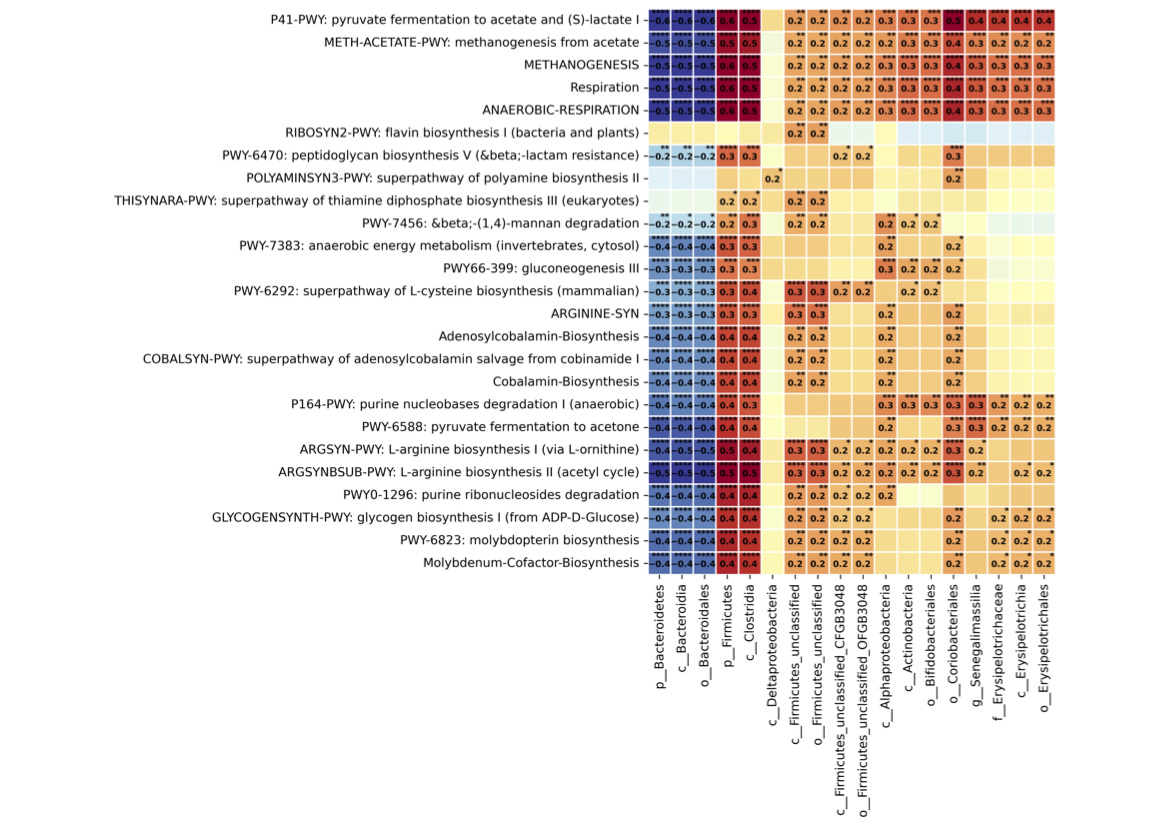

Supplement: Supplementary file 3 [file Image1.tiff]
